# Abstracts and Gleanings

**Published:** 1888-01

**Authors:** 


**A Substitute for Iodoform.**—The disagreeable odor of iodoform has always been one of its greatest drawbacks. Its numerous combinations have found their several fields of usefulness, and of late iodoform collodion has become popular. Sub-iodide of bismuth is considered a rival to iodoform, and has not the drawback of a disagreeable odor. Judging from the usefulness of iodoform collodion, it has occurred to us that a sub-iodide of bismuth collodion might be of service where iodoform collodion fails or cannot be used on account of the odor. We should be glad to have our friends among the physicians give this collodion a trial. —*St. Louis Drug Reporter*.

### ABSTRACTS AND GLEANINGS.

**The Crowning Achievement of Ophthalmic Surgery of the Present Century.**—Under this title Dr. L. Webber Fox describes in the *Medical and Surgical Reporter*, Professor von Hippel's operation for the transplantation of the rabbit's cornea to that of man. By this operation Von Hippel has restored sight to an eye practically blind, and Dr. Fox predicts that it has a brilliant future in doing this kind of work. He saw the patient upon whom the first successful operation was performed, and learned its technique. We cannot do better than to furnish our readers with Dr. Fox's very clear and complete description.

The patient operated upon was a young, healthy girl, nineteen years of age, who suffered with a leucoma of the cornea of the right eye, obscuring vision to such an extent that qualitative perception of light only remained. The leucoma simplex obscured the central portion of the pupil, leaving, however, a circle of clear cornea at its outer margin, probably two mm. wide. The instrument devised by Professor von Hippel for performing this operation is most ingenious. The trephine is driven by a clock work and the cylinder is graduated so as to regulate its cutting depth in the cornea. The leucomatous cornea opposite the pupil was removed by this instrument. The eyelids were separated by an ophthalmostat; then, under the influence of cocaine, the trephine was placed on the cornea perpendicular to its plane, the clyinder so graduated as to cut a certain depth, 0.7 to 0.9. This cylinder is then put in motion by a spring clock motion much after the manner of a Dudgnon's spygmograph; the hand simply steadies the instrument against the probe; after the circular incision is made comes the most important and delicate part of the operation, *e. i.*, the dissection of the leucomatous tissue from the Decemet's membrane. This is done by grasping the inner lip of the incised tissue, and with the greatest care and precision this tissue is removed down to the basement membrane lying in juxtaposition to Decemet's membrane. If, after the removal of this circular piece of cornea, it is found that Decemet's membrane bulges forward through the circular opening, which in almost every case it does, a paracentesis of the anterior chamber is made at the corneoscleral margin, relieving the intra-ocular pressure.

The rabbit selected from which to obtain the graft is a young, healthy doe. The eye, which is thoroughly cocainized, is drawn forward by an assistant who inserted under the superior and inferior recti muscles two strabismus-hooks; the eyelids are kept open with an ophthalmostat; the drawing forward of the globe enables the trephine to be inserted and watched more accurately in its incision; the cut is made through cornea and Decemet's membrane; this graft is then inserted in the incision made in the patient; a fine probe running through the cylinder of the trephine is pushed downward, forcing the graft into place; after the removal of the trephine the upper eyelid, which is drawn forward and downward, is pressed against the inlaid tissue, all being held firm by a pressure-bandage, delicately adjusted, and the patient, of course, lying on his back. After three days the bandage is removed, and the eye examined. If the grab is *in situ*, it will probably be somewhat hazy; if the edges have not turned up, a successful result may be prognosticated.

Dr. Hippel recently showed a second patient and demonstrated his operation before the Ophthalmological Society of Heidelberg. The patient was found to have a visual acuity of 20-200, and read Jaeger's No. 6, from which it would be inferred that the new cornea did not clear up completely.—*N. Y. Medical Record.*

**Indolent Ulcers.**—A writer in the *New England Medical Monthly* says:

Mr. A. B., aged 58. laborer, and at times a hard drinker, came to me with the most formidable ulcer of the leg I have ever seen. The limb below the knee was very much swollen and œdematous, and the ulcer measured six and one-half inches in length and five in width; the edges were abrupt and hard, with a depth of one-half to three-fourths inches. At the external margin a slough was forming, and the surface of the ulcer was hard and grizzly, without even an attempt at granulation.

The patient was put to bed and his clothes removed from the room with orders that they should not be brought to him without my consent. Hot flaxseed poultices were applied to him day and night every two hours four days, at which time the ulcer presented a very much altered appearance. The slough had separated and come away, the hard, indurated edges were soft and yielding, the dark, offensive pus had disappeared from the floor of the ulcer, and a more laudable pus taken in its place; yet there were no signs of granulation.

The wound now was the size given above, and the next dressing was as follows: Common crystalized boric acid was powdered in a mortar until the crystals were broken as finely as possible without reducing it to a powder, and the wound was completely filled with it. Several large handsful were used until the surface of the skin was reached; over the acid was sprinkled iodoform, but this was discontinued in the other dressings. A snug bandage was applied, and the patient left to his own meditation for a period of four days. When about to remove the bandages, I confess to some misgivings as to the condition I should find the ulcer in, but to my gratification the change was wonderfully satisfactory. The depth was reduced at least one-half, and the granulations abundant and healthy, while around the edges little peninsulas of new skin were pr jecting toward the centre. The boric acid had entirely disappeared (where I do not know) and the amount of pus present was only sufficient to render the bandage easily removable. Three dressings as before at intervals of four days, and the level of the surrounding skin was reached, and the dimensions of the ulcer were diminished more than one-half; the acid was discontinued and an ointment composed of

℞ Iodoform........................................gr. xx

Bismuth sub carb...........................ʒ j

Cerat simp....................................℥ j

M.

was applied twice daily until the twenty-first day, when the skin formation was complete and the patient allowed to put on his clothes.

The skin was kept soft with oil for a week or two longer, when the patient was discharged, cured. Three years later the case was still complete, and there were no signs of its recurrence. The third and fourth applications of the acid gave the only pain complained of, and then it was so severe as to require a dose or two of morphia.

This treatment has been employed in a number of cases subsequently, and the results in every case have been equally satisfactory. The main points are:

1st. Rest in the recumbent posture.

2d. Poultice until all sloughs are disengaged and the walls softened.

3d. Fill the cavity with the boric acid and do not repeat oftener than three or four days. Continue until the granulations are satisfactory.

**The Treatment of Acute Bronchitis in Children by Antipyrin.**—Friedlander, in the *Therapeutic Monatschriften*, quoted editorially in the *Journal of the American Medical Association*, October 15th, 1887, states that before using antipyrin he lost many children who were attacked with this disease, and its duration was from two to three weeks. Since using it the mortality has been wonderfully diminished and the average duration reduced to one week. If the cases are classified according to their amenability to antipyrin, two groups must be formed, the first embracing those that are benefitted by the drug, the second those that receive little aid from it. The first group embraces those who have a temperature of 104° F. (40° C.) and who are well nourished; the second those that have a moderate temperature, 102° F. (39° C.) or less, and who are not nourished. Both classes are aided by antipyrin treatment, but the former much more so, as is shown by the following statistics. In a recent severe epidemic of acute bronchitis among children in his neighborhood, he, as well as his fellow-physicians, lost 50 per cent. of their patients; but since he has used the antipyrin treatment he has lost none belonging to the first category, and the duration of illness has averaged not more than a week, and the percentage of loss in the second category is reduced to 10. He suggests that the reason why the drug does not apparently accomplish as much good in weak children is because they are not able to withstand its unpleasant side effects, such as the profuse sweating that it induces. Usually, after the administration of a dose, the little patients break out in a profuse perspiration, the temperature falls, they sleep for an hour or so, and awake with a feeling of being well. The cough is usually looser and the breathing easier.

The dosage recommended is for children under five and over two years, 12 or 13 grains (0 9 gms.) Its effect will continue for from twelve to fifteen hours, and therefore two doses must be given daily. To a child a year old, or less, 7 grains (0.5 gms.) may be given, but only one dose will be needed in twenty-four hours. During convalescence one half of the above mentioned quantities are given at a time. The treatment of acute bronchitis among children by antipvrin, as recommended by Friedlander, does not exclude the simultaneous use of expectorants.—*College and Clinical Record.*

**The Pathogenesis of Pterygium.**—Dr. Theobald (*Epitome*): The generally accepted theory of Arlt, that pterygium has its origin in a marginal corneal ulcer, to which a tag of conjunctiva has become attached, he thought was unattenable, because if this were its usual mode of origin pterygium would be found approaching the cornea from everv possible direction, since marginal cornea ulcers are not apparently more frequent in one position than in another. It is known, however, that such is not the case, but that pterygium is almost always situated directly over the recti muscles, and that in a very large proportion of cases it is over the rectus internus. The more recently proposed theory of Poncet, that pterygium is due to the presence of microbia, which turned their way under the corneal epithelium, is open to the same objection, for this also assumes the existence of a precedent corneal ulcer. The view long held, that conditions which tend to induce chronic hyperæmia of the conjunctiva favor the formation of pterygium, he thought was well established.

Assuming that this view is correct, are there reasons why a localized hyperæmia of the conjunctiva should be of frequent occurrence where pterygium usually forms to the nasal side of the cornea? This was answered affirmatively. The close connections between the vessels of the recti muscles and those of the anterior portion of the conjunctiva were referred to, and it was pointed out that the determination of blood to these muscles might influence the blood-supply of the overlying conjunctiva, and that this would be the case especially with the recti interni, since they were the largest of the straight muscles and in close relationship with the conjunctiva, because attached to the sclerotic nearer to the corneal border than any of the others. Abnormality in the distributing of the blood-supply of the internal recti muscles, and of the overlaying conjunctiva, and, more frequently still, disturbance in the normal relationship between convergence and accommodation, such as insufficiency of the internal recti muscles, the different varieties of the ametropia—these were regarded as the usual causes of pterygium through the localized hyperæmia of the conjunctiva to which they give rise.

**Cascara Sargrada and its use in the Treatment of Constipation.**—Dr. John W. Farlors, M. D., Boston, Mass., writes (*Epitome*): According to my experience, the cases for which cascara is particularly adapted are the chronic cases and especially those with weak and atonic digestive organs. For such patients it is far superior to rhubarb, senna, aloes, licorice powder, and the usual laxatives, either alone or in the various combinations. For acute cases its peculiar qualities are not so much required, although it generally acts promptly, surely and without secondary constipating effect.

I have generally used the fluid extract, for several reasons. It is an active and reliable preparation, the dose is small and can be easily regulated by increasing or diminishing the number of drops taken at a time. The taste is bitter, to which some object, while others find it not unpleasant. To the latter it can be given in water or with equal parts of glycerine and water. I should say that the fluid extract of licorice is perhaps as good a recipient as any. Cascara cordial has an agreeable taste, and is preferred by many. The dose, is, of course, larger and is not so easily regulated as the flnid extract. The solid extract is given in pill form, and consequently can be tasted. If, however the dose in each pill proves too large, a lot with a smaller dose has to be procured, which is a disadvantage as compared with the fluid extract.

The dose of the cordial is about a teaspoonful morning and night, or oftener. The solid extract is given in doses of three grains or less three times a day. The dose of fluid extract is from five to twenty-five drops, and I generally order it to be taken as follows: If the case is of long standing and one in which many drugs have been tried, I direct ten or fifteen drops to be taken in water before each meal and at night. If that does not cause one soft dejection a day, in two or three days I increase the dose to twenty-five drops four times a day, and tell the patient to take sufficient to have one dejection a day.

**Treatment for Carbuncle.**—Dr. H. O. Pantzer (*Indiana Medical Journal*) writes: Dr. Bidder employs a three-per cent. solution of carbolic acid, and injects from one to four grammes, varying from one to two centimeters from the center of the carbuncle, not more than four points of injection being necessary for the carbuncle of half the size of an adult hand. The injection need not be repeated ordinarily, and is followed up by the application of adhesive plaster over the carbuncle. Dr. Bidder says, in substance, obviously the introduction of the needle is painful, but immediately there follows a pleasant sense of painlessness. After the lapse of a few hours of spontaneous burning and pricking in the carbuncle occurs, but is of transient duration, and on the day following the fever abates and good feeling reestablishes itself. The subcutaneous infiltration rapidly vanishes, and but little secretion is discharged from the fistulous opening, which closes with healthy granulations on the third or fourth day. The advantages of this method lie in the safety and rapidity of the method, and the least annoyances for the patient, who requires no poultices, and can pursue his every-day occupation. Besides, no ugly scar is left, as after the old-time crucial incision, a matter of great moment where the carbuncle infects the face or neck.

If Dr. Yarr's investigation may be relied on, and the staphylococcus pyogenes aureus must be regarded as the pathogentic factor in producing this form of inflammation, then the explanation of this method of cure is simple, and rests in the germicide properties of the carbolic acid injection used.

I trust this communication may be of interest to those of your readers who have not access to the paper from which I quote.

**Indications for the Use of Nitro-Glycerine.**—The value of nitro-glycerine in various diseases, as angina pectoris, hemicrania, and neuralgia, and also in sea-sickness, certain forms of anæmia, etc., depends on the existence in these of an irregular distribution of the blood. This abnormal condition may be recognized by a certain grade of pallor of the skin, especially of the face, an appearance co-existent with a weak pulse and small radial arteries, hard and frequently situated at a certain depth. When, on the contrary, the headache and neuralgia occur in persons with chronic congestion of the subcutaneous vessels of the face, nitro-glycerine is contra-indicated ; and similarly it should not be used in asthma when the face is conjested from the effects of the emphysema. Thus it may be said that the best therapeutic results from nitro-gycerine may be obtained in these cases in which angina pectoris, neuralgia, etc., are associated with pallor of the countenance.

The condition of the pulse is the best indication for the use of nitro-glycerine, and the safest guide for the determination of the time in which should begin the cure. The smaller the radial artery is, so much more rapidly does it dilate under the influence of the drug, and so much the less are the secondary effects produced by it ; on the contrary, the f ller the pulse, and the more tense the radial artery, so much the less this resents the influence of it.

When the pulse is small, the usual dose of one drop of one per cent. solution is sufficient, while if the pulse is large, two drops may be required to obtain the full effect. When the radial is soft and the pulse weak, smaller doses should be given—½ to ¼ of a drop. The sensation experienced by the patient, throbbing and pain in the head, as well as the distention of the radial artery under the observer's finger, should be the guide for the increase of the dose.—*Giornale Internazionale delle Scienze Mediche.*

**Veratrum Viride.**—Dr. A. Ady, Muscatine, Iowa, (*Medical and Surgical Reporter*) says: I have been using it for thirty-four years, and it has disappointed me fewer times than any other medicine. In proper doses, I do not believe that it has any tendency to weaken the heart. On the contrary, by slowing its contractions it gives that organ time to rest and recuperate. As a heart tonic I consider it equal, or superior, to digitalis; as a controller of the heart and arteries, superior to all other remedies in use.

In all other inflammatory disorders in which the action of the heart is too strong and frequent, it is superior to blood letting. In acute pleurisy and pneumonia, when a patient is brought thoroughly under its influence, he is about cured. The cumulative effects of veratrum viride are not dangerous, as they are relieved by vomiting, after which the patient will almost invariably ask for something to eat.

In typhoid fever veratrum viride is one of the best remedies. Thoroughly unloading the stomach and portal circulation, controlling the circulation, preventing the tongue from becoming dry and parched. I feel sure, also, that in many cases it has shortened the course of the disease. Patients eat well while taking veratrum viride. The doses in these cases must be small, 1 drop of Norwood's tincture every four hours being often sufficient.

In puerperal convulsions it is a most efficient remedy, but must be given in very large doses, the uræmic condition producing tolerance of its effects. A patient in puerperal convulsions can safe y take from 30 to 60 drops of the fluid extract. I have not been obliged to repeat the dose in such cases, nor has vomiting occurred.

In membranous croup it is *the* remedy; but must be given for effect, not in ordinary doses. I have given a child two-and-a-half years old, 20 drops of the fluid extract inside of six hours, with the best results. It is worse than useless in diphtheria, scarlatina, and pyemia.

I have used it in small-pox, bringing the patient under its influence in the initial fever, and not allowing the pulse to rise above sixty beats per minute during the course of the disease. In this case no delirium occurred, nor any secondary fever, and when the crusts dropped off no pitting of the skin was left. Dr. Maxwell, of Davenport, now dead, confirmed my experience, after ample opportunity to test it in the army pest-houses during the war. He also claimed that, if the drug was administered during the course of vaccination, no scar would be left.

The only unpleasant symptoms, which I have witnessed in the use of veratrum viride, have been nausea and vomiting, and according to Prof. H. C. Wood (*American Medical Journal of Science*), they can be entirely avoided by separating the alkaloid containing the sedative principle from that of the emetic, as was done by Mr. Chas. Bullock, of Philadelphia, with the article experimented on by Prof. Wood.

I have been trying for the last fifteen years to get some pharmacist to prepare and put the viridæ sulphas upon the market. It certainly would be extensively used, especially by the practitioners of the West.

In conclusion, I would say that, if I had to rely upon four articles for an armamentarium, they would be quinine, calomel, opium, and veratrum viride.

**Injection for Hemorrhoids.**—Dr. O'Neal (*Medical Summary*) uses the following fluid: ℞. Carbolic acid, glycerine, water, each ʒij.; tannic acid. ʒj. M. From 3 to 8 drops injected into the tumor. Not to be used while inflamed; not to inject more than two tumors at one operation. The pile sac disapears in a week or ten days without causing a slough or pain productive of any inconvenience, and another may then be operated on.—*American Medical Journal.*

**Hints in the Treatment of Gonorrhœa.**—Dr. F. N. Otis offers the following points in the treatment of gonorrhœa:

1. Fully explain to the patient the inefficiency of popular remedies, and the dangers attending their use.

2. Secure the absolute personal cleanliness, thereby preventing infection of other parts, and insist upon as nearly perfect rest in bed as the exigences of the case will permit.

3. Soak the penis frequently in water as hot as can be borne, but more especially during the act of micturition.

4. Recommend milk as a diet, and prescribe alkaline diuretics and mineral waters as internal medications.

5. Secure absolute freedom from sexual intercourse and from thoughts associated there with. Perfect faith in and obedience to these simple formulæ he insists will insure a successful ending of all uncomplicated cases before the beginning of the seventh week. —*Practice.*

**Electricity as a Pain Killer.**—Dr. F. T. Paine (*in Daniel's Medical Journal*) writes: I had an opportunity yesterday to establish, to my own satisfaction, a theory I have long entertained, viz: that impressions made upon the peripheral terminations of the different nerves by the Farradic current, are entirely different, diametrically opposite—they are the antitheses, the antipodes, (so to speak), the antagonists, *and the antidote* to all painful impressions, made by any other means whatever!

My daughter, while preparing b'eakfast, scalded her wrist so as to take off the epidermis The only application was the Farradic current, through a water bath, powerful enough to contract the muscles most violently, and the pain of the burn was INSTANTLY RELIEVED, her cooking was resumed, and breakfast was delayed only thirty minutes. It is now thirty-six hours since the scald, and although the skin is removed, she has not had a moment's pain.

The interest is not in this single case, but in the rationale; how is the pain relieved by, what the Erb calls "the irritant *par excellence.*"

I am aware that all I have published is enigmatical and paradoxical to the profession and to the laity. I only need an opportunity to make it plain."

**Enlarged Tonsils.**—Dr. Stallard, of Colorado, holds that in enlarged tonsils there is very frequently an enlargement of the liver, which has a great deal to do with the enfeebled health of the patient, and he further thinks that the great amount of *sweets* consumed by children derange the whole glanular system and contributes largely to the number of cases of chronic enlargement of the tonsils that we meet with. The writer is satisfied that the excessive use of sweetmeats in the form of candies, etc., is quite injurious to the health of children and the cause of much poor health. Deafness often attends enlarged tonsils and is generally due to adenoide growths in the pharynx pressing upon eustachian tubes.—*Medical Summary.*

**A Valuable Formula in Menstrual Irregularities.**—Menorrhagia, metrorrhagia, dysmenorrhea and amenorrhea are terms designating uterine conditions, common, yet often very intractable, exhausting the strength of the patient, and the patience of the physician in their persistency.

If thoughtfully considered, these conditions are quite amenable to treatment in nearly every case.

Mrs. S., always regular in time and quantity of menstrual flow, was obliged to do a prodigious amount of work in the care of her only sister, to whom she was very much attached, and who had been taken suddenly and dangerously ill. The over-work, coupled with the anxiety and sorrow at the sudden death of the sister, brought on a metrorrhagia which seemed incontrollable. The menses were excessive, amounting to flooding, continued from eight to ten days, then ceased for a week to return with increased severity. Ergot, erigeron, cinnamon, alum, gallic acid and other astringent measures did not produce an impression upon the disorder. The patient became exhausted and anemic. The following prescr ption was finally advised and the patient was kept quietly in bed for one week:

℞. Fl ext. senecio aurens.........................
Fl. ext. aletris farinosa.......................aa℥ iij,
Fl. ext. cimicifuga racemosa...................
Fl. ext viburnum prunifolium...............aa℥ v.
M. Sig.—Ten drops in water every three hours.

The flow soon ceased and did not return for a month. Then in proper quantity and time and has since been very regular.

The above prescription seems to act more readily where the disturbance is of nervous origin, or is a result of nerve exhaustion.

In dysmenorrhea, gelsemium has been added in place of the cimicifuga, and very excellent results obtained. In amœenorrha the prescription may be accompanied or alternated with iron in some form. In sub-involution, following confinement, the combination produces excellent results. In many cases it must be persisted in as it may not effect a cure immediately, but it is generally prompt and satisfactory in its action. In old standing cases of "falling of the womb," the pain and distress, the back-ache and dragging sensations will be quickly relieved by this combination. The writer has used it for several years with excellent resu ts and speaks from wide experience. The remedies are unpleasant of administration, but the smallness of the dose commends it to the physician.—*Chicago Medical Times.*

**A New Method of Treating Diseases of the Skin Locally.**—Dr. Knagg (International Medical Congress, 1887), proposed as a substitute for ointments the employment of emulsions which drying on the skin form protective films. He had employed these dressings for the past two years with gratifying results in eczemas and other non-specific exudations. The inunction of the body with fixed oils has been found of immense service, but they are not adhesive, and exuding fluids readily escape from the surface. There are two methods of rendering oily substances adhesive: 1. By adding to them resinous gummy, or alkaline substances. 2. In making use of gums to combine or emulsify a fat with water.

Adhesive preparations, unlike oils, tend to arrest skin-action. Thus tar, varnish, or c llodion form an impervious covering. Ointments to which adhesive substances are added diminish skin-action, but do not abolish it.

Well-made emulsions resemble milk or cream, are soluble in water, a vegetable gum, and a suitable antiseptic.

Thus the formula preferred would read:

℞. Paraffine molle.......................... ℥ j,

Pulv. gum acac.......................... gr. clx,

Boracic acid.......................... gr. xvi,

Aquæ, ad.......................... ℥ ij.

Stir until emulsified.

Bismuth, zinc, sulphur, or other medicament may be added as desired. Smeared over the skin, a film is formed, which is flexible and protective, and the antiseptics and medicaments have the best opportunity of exercising a beneficial influence.

The author called especial attention to the fact that all lint and textile dressings could be dispensed with. He did not expect these gum emulsions to displace older methods, but that they would serve a useful purpose.—*The Epitome.*

**Infantile Constipation.**—A very successful remedy for this is podophyllin in small doses; iridin may be combined with it with good effect. Make a tincture of the following:

℞. Podophyll..........................
Iiridin.......................... aagrs. v,
Spts. ammon. arom.......................... ℥ j.

Digest for several days and filter. From 1 to 2 drops of this may be combined in mixture along with syrup of orange. This is the dose for a child of one year and under.—*New York Medical Record.*

**Phosphate of Sodium Hypodermically in Phthisis**—Baratous claims good results from the following:

℞. Sodii phosphat.......................... part i,
Sodii sulphat.......................... part i.
Aquæ destill.......................... parts xv.
M.

Filter before using. Injections of from 45 to 90 minims may be made once or twice weekly, in the sacrolumbar region. These injections are followed by fever, cerebral excitation, and a feeling of fatigue for one or two days. A feeling of general improvement follows, the appetite returns, perspiration lessens, and expectoration loses its purulence. Gain in weight has been observed. *Revue de Therapeutique*, July 1, 1887.

**The Treatment of Morphiomania.**—Professor Ball states that in the Asylum of St. Anne it has been found impracticable to treat morphiomania by complete and sudden suppression of the drug. The method found most certain and satisfactory was by gradual suppression. Insomania and nervousness were combatted by bromide and chloral. Heart symptoms were treated by sulphate of sparteine in doses aggregating three (3) grains *per* diem. The dangers of complete and sudden suppression, aside from the tortures thus entailed, are very great—much greater than the great body of practitioners are aware of. In a case cited by M. Ball, that of a woman who took from 15 to 20 grains daily of hydrochlorate of morphine, abrupt and total suppression was followed by collapses which necessitated immediate use of the drug. She was then put upon the gradual treatment, and for six weeks she enjoyed perfect health. Just when the morphine had been reduced to a minimum and was about to be withdrawn altogether, the patient fell into a state of collapse and suddenly died. The autopsy revealed fatty degeneration of the heart, with incipient degeneration (fatty) of the muscular fibres, and of other organs, while the spleen, the liver, kidneys and nerve centres were surcharged with morphine.—*St. Louis Medical and Surgical Journal.*

**Nitrate of Potash as an Emetic.**—Dr. Moore (*Medical and Surgical Reporter*) says: I wish to call the attention of the profession to the value of nitrate of potash as a remedy for vomiting.

Notrate of potash, properly administered, is one of the surest remedies we have for the relief of vomiting. I have been using it as an anti emetic in my practice, for the past twelve years, with the happiest results. I have made known its value in this respect to a number of my medical brethren, and they have each expressed themselves as pleased and surprised at its power in allaying nausea and checking vomiting. The manner of administering it is as follows: Give $\frac{1}{4}$ of a grain, dissolve in a tablespoonful of cold water, every four or five minutes until the vomiting is relieved; which will usually be in from ten minutes to half an hour. I give it in all cases of vomiting, except reflex, and that due to irritant poisons, and have found it succeeded in ninety per cent. of my cases. Try it; you will be surprised and pleased. It is pleasant to take, rendering it more valuable in nausea and vomiting in children.

**Ague-Cake.**—For ague-cake Professor Bartholow advised a combination of Squibb's aqueous extract of ergot, arsenite of iron and salicylate of cinchonidine, to be taken three times daily. Locally employ ung. hydrarg. oxidi rubri.—*College and Clinical Record.*

**To Extract a Tooth Without Pain.**—Dissolve one grain of cocaine hydrochlorate in eight minims of three per cent. solution of carbolic acid ; inject about five minims of this into the gum on the outer side of the tooth, the remainder into the inner gum ; wait five minutes and extract the tooth.

**Treatment of Sciatica**.—Some months ago I got my druggist to make tablet-triturates, each of which contain 3 drops of the following mixture:

R. Tincture of aconite root.... .
Tincture of seeds of colchicum..
Tincture of belladonna........
Tincture of actea racemoso....aa equal parts by volume.

Of these, I give one every four, six, or eight hours, according to the necesity of the case. It takes not long to impress the system, especially with aconite, nor does it, as a rule, require more than three days to make the patient so firmly believe in its efficacy that he has no need of persuasion to insure its continuance until complete relief.

In neuralgia of the auxillary and brachial nerves, it has proved quite as efficacious as in that of the great crural branch —*Analetic.*

**Cascara Sagrada.**—In the *Therapeutic Gazette*, for March, 1887, the writer gave his experience with the above drug in fifty carefully observed cases with the following results:

Of the fifty cases the drug was useful in forty-three (43). It entirely failed in seven (7). In the forty-three favorable cases the dose of the drug was diminished in twenty-nine instances after using it for a longer time. In *no* instance was found necessary to *increase* that dose which by trial had been found sufficient to cause a daily evacuation of the bowels.

The fluid extract was the preparation used, in 20 drops doses three times a day.

Continued use of the drug, both in private and dispensary practice, tends to strengthen the writer's opinion as to its value as a laxative in chronic constipation, and enables him to recommend it as a valuable addition to our armamentarium.

Medical Dispensary, University Hospital, November 18, 1887.

**Iodide of Sodium.**—Iodide of sodium is considered by Dr. Richardson as a valuable substi ute for, or adjunct to, iodide of potassium. In chronic eczema and painful rheumatic affections it often answers well when iodide of potassium does not agree with the digestion. Combined with arsenic it is useful in lepra and psoriasis. Externally applied, in cases of indolent ulcer, chronic syphilitic sores, and offensive discharges from the nostrils, it acts as a good antiseptic. The following is given as a formula for the purpose:

R. Sodium iodide................................℥ ss,

Tincture of myrrh................................ʒ i,

Rectified spirit................................℥ ii,

Distilled spirit................................℥ vi.

To make a solution of eight ounces. Used in the form of a fine spray from Seigle's spray-inhaler, Dr. Richardson found it of the greatest service in a case of syphillitic ulceration of the fauces.—*Therapeutic Gazette.*

**Biliary Calculus.**—M. Dujardin Beaumetz recommends that persons suffering from biliary calculus should be enjoined to abstain from all fatty substances and carbo-hydrates which may furnish cholesterine. Peas, in particular, should be forbidden, as they contain a fatty substance similar to cholesterine; the excessive use of meat must be avoided, and eggs should rarely be eaten. A mixed diet composed of meat and green vegetables may be eaten, and also potatoes. Fruit may be taken if not too sweet; pastry should be forbidden. Meals should be taken frequently in order to keep the gall-bladder at work. Wine mixed with Vichy water makes a good drink. The bowels should be kept open and plenty of exercise should be taken.—*British Medical Journal.*

**A Large Dose of Croton Oil.**—Not long since I was waiting on a man who had malarial fever complicated with a troublesome diarrhœa, which was readily controlled with camphorated Dover's powders, and as soon as they stopped he (like the Dutchman) wanted them to be moving again, and decided to take a dose of castor oil by the family. Consequently, he took a tablespoonful of it, and in less than five minutes he was seized with violent purging and vomiting which alarmed them, and the bottle was examined more carefully and found to contain an irritating liniment composed of equal parts of olive and croton oil.

This is the largest dose of croton I ever heard of being taken, and yet it did not prove fatal.—*Dr. Owen in Medical Brief.*

**Sensible.**—The *Medical Age* says: It is reported of an intelligent man who settled in a strange town and who wished to secure a medical adviser for his family, that he went to the postmaster to find out which of the local practitioners took the most medical journals. His reasoning was that the man who takes the most journals of his profession, is well read and up with the times, and consequently the one whom any sensible man would prefer to employ. Unquestionably this reasoning is sound, but does not go quite far enough. He should have discovered which of the doctors pays his subscription to the journals most promptly. It is the man who promptly at the beginning of the volume transmits his subscription, that is the man to tie to. Such men are the pride of the profession and are always safe counsellors.

**Phosphate of lime** is strongly recommended by Dr. Rebory for night sweats of phthisis. M. Potain, and after him Dr. Rebory, employ the tricalcic phosphate in doses of 4 grammes, often necessarily increased to 8 and 15 grammes The excellent results obtained are attributed by Dr. Rebory to some special action of the medicine upon the perspiratory apparatus. It would seem more likely that the general improvement in the condition of the patient brought about by the phosphate should be the reason for the diminution of the night-sweats one of the symptoms most indicative of the great debility of persons subject to phthisis.—*Philadelphia Medical Times.*

